# Emerging epidemiology of invasive group a Streptococcus in tropical Australia: a 21-year population-based data linkage study

**DOI:** 10.1007/s15010-026-02904-w

**Published:** 2026-08-25

**Authors:** Himali Erandathie Ratnayake, Damon Eisen, Oyelola Adegboye, Anton Pak, Emma McBryde

**Affiliations:** 1 College of Medicine and Dentistry, James Cook University, Townsville, QLD Australia; 2 Menzies School of Health Research, Charles Darwin University, Northern Territory Darwin, Australia; 3 Public Health and Tropical Medicine, College of Medicine and Dentistry, James Cook University, Townsville, QLD Australia; 4 Centre for Business and Economics of Health, The University of Queensland, Brisbane, QLD Australia; 5 Australian Institute of Tropical Health and Medicine, James Cook University, Townsville, QLD Australia; 6 Burnet Institute, Melbourne, Victoria Australia

**Keywords:** Invasive group A Streptococcus, *Streptococcus pyogenes*, Infectious diseases, Tropical Australia, Sepsis

## Abstract

**Purpose:**

Invasive Group A Streptococcus (iGAS) is an important cause of severe bacterial infection with increasing incidence and substantial socioeconomic disparities. This study quantified long-term trends, spatial distribution, and determinants of iGAS incidence in Northern Queensland, Australia.

**Methods:**

A population-based retrospective cohort study was conducted using linked hospital data linkage from 2000–2020. Incidence rates were calculated per 100,000 person-years and temporal trends were assessed. Spatial clustering was evaluated using Moran’s I statistic for spatial autocorrelation. Determinants of incidence were examined using a negative binomial generalized additive model (GAM).

**Results:**

A total of 933 iGAS events were identified among 870 individuals. Incidence increased substantially over time, with the highest rates observed among older adults and a secondary peak among infants. Age-standardised incidence was approximately nine-fold higher among Indigenous compared with non-Indigenous populations for both males and females. Spatial analysis demonstrated significant clustering of incidence (Moran’s I = 0.19, p < 0.001), with the greatest burden occurring in remote and very remote regions. In multivariable analysis, Indigenous status (IRR = 8.97, p < 0.001) and male sex (IRR = 1.28, p < 0.001) were independently associated with higher incidence. Smooth terms showed significant nonlinear effects of age (edf = 7.45, p < 0.001) and calendar year (edf = 8.04, p < 0.001), indicating heterogeneous age patterns and temporal acceleration in incidence.

**Conclusion:**

iGAS incidence in Northern Queensland increased markedly over two decades and was characterised by pronounced geographic and Indigenous health inequities, highlighting the need for targeted prevention strategies in high-risk populations and regions.

**Supplementary Information:**

The online version contains supplementary material available at https://doi.org/10.1007/s15010-026-02904-w.

## Introduction

Invasive Group A Streptococcus (iGAS) infections are serious conditions with high morbidity and mortality. Clinical manifestations specified as invasive are sepsis, bacteraemia, bacteraemic pneumonia, necrotizing fasciitis (NF), Streptococcal Toxic Shock Syndrome (STSS), arthritis and severe skin and soft-tissue infections (SSTIs) [1, 2]. Every year, at least 663,000 new cases of iGAS are reported worldwide, with estimated 163,000 deaths [3]. Hence, iGAS is recognised as a significant global health problem and is listed among the top 10 infectious causes of mortality [4].

iGAS represents a significant burden on the Australian healthcare system, consistent with global patterns of morbidity and mortality associated with the condition [5, 6]. As of 1 July 2021, iGAS was designated a nationally notifiable disease in Australia, facilitating improved surveillance and reporting across the country [7]. The incidence of iGAS varies significantly across different regions in Australia, likely influenced by regional disparities in comorbidities, socioeconomic status, and climate [8]. Notably, Indigenous people experience disproportionately higher rates of iGAS, a disparity thought to be associated with higher prevalence of chronic health conditions and social determinants such as low socio-economic status and overcrowded housing [6, 9]. The disease also appears to be more prevalent in tropical climates, suggesting a potential environmental component to transmission [6]. Longitudinal studies conducted in Australia revealed an increasing incidence of iGAS over time [6]. Within this context, Queensland has emerged as a key hotspot, bearing a substantial proportion of the national iGAS burden. According to the National Notifiable Diseases Surveillance System (NNDSS), Queensland recorded the highest number of reported iGAS cases nationally in 2024 [10].

Against this backdrop, the present study aimed to estimate the burden of disease and describe the epidemiology and associated risk factors for iGAS in a region of North Queensland, Australia. We utilised a large, longitudinal hospital data linkage spanning 21 years to conduct this study. To the best of our knowledge, this represents the largest study of iGAS in North Queensland using linked hospital data to date.

## Methods

### Study setting and study period

**Fig. 1:**
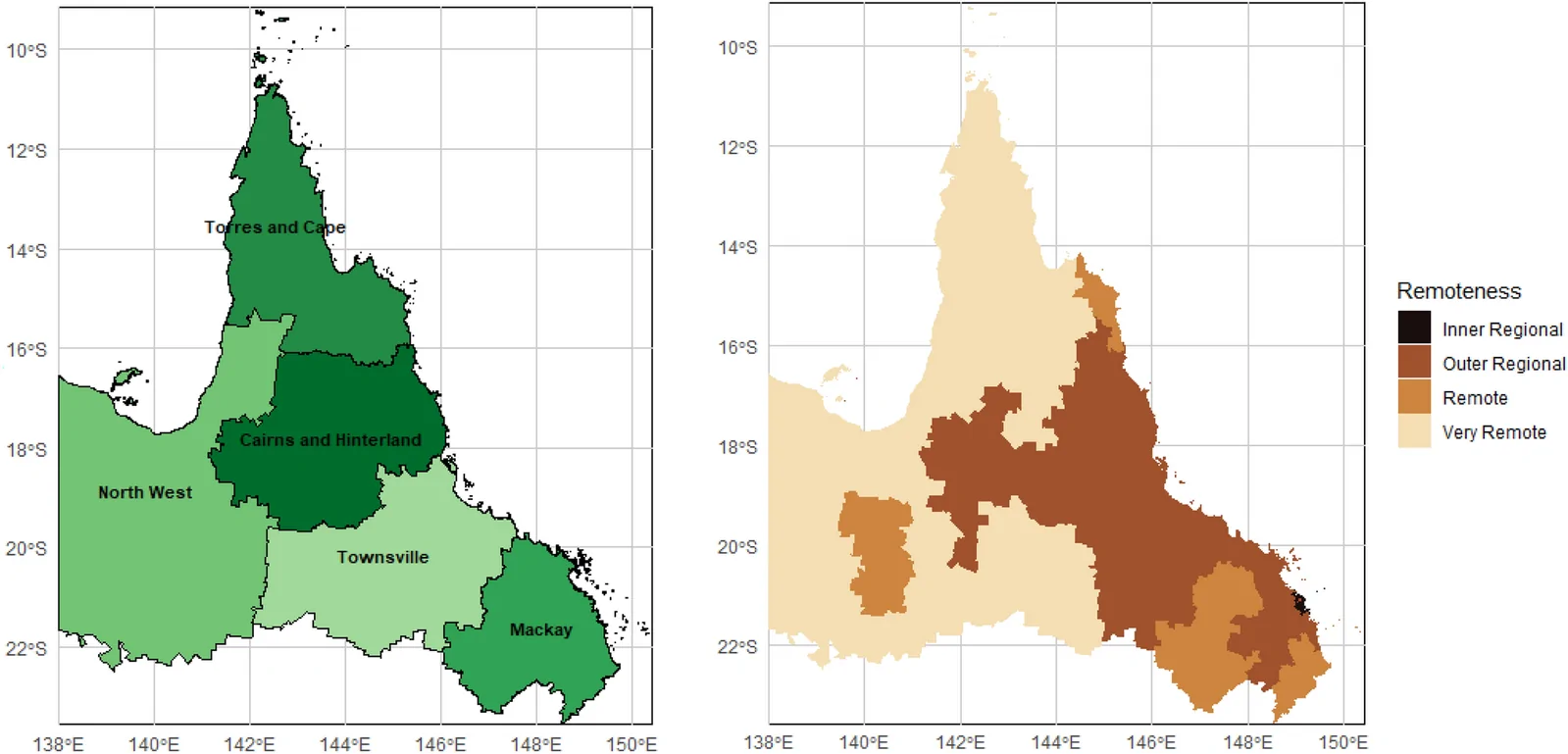
Geographic location and remoteness classification of the study area. **A**. Hospital and Health Service areas included in the project. **B**. Australian Statistical Geography Standard (ASGS) remoteness structure classification of the study area (2021)

This study was conducted in five Hospital and Health Service areas (HHSs) in Northern Queensland namely, Cairns and Hinterland HHS, Mackay HHS, North West HHS, Torres and Cape HHS and Townsville HHS (Fig. 1A). The period of data collection was from 1 January 2000 to November 2020. This region is recognized as a tropical area, characterized by its warm, humid climate. According to the Australian Statistical Geography Standard (ASGS) Remoteness Structure, this region contains no major cities, with most of the population living in outer regional (63%) [11] (Fig. 1B). Indigenous community contributes to 13.3% of the population [11].

### Data sources

The data for this study is obtained from a hospital data linkage from year 2000 to 2020 using Queensland Hospital Admitted Patient Data Collection (QHAPDC) and Emergency Data Collection (EDC). This data linkage includes data on all admitted patient episodes in both public and private hospitals. Details regarding the original database, including the constituent sources and the linkage methodology, are described in Eisen et al. [12], which initially included only the Townsville Hospital and Health Service (HHS). The current study utilised an expanded version of this linked database, incorporating data from four additional HHSs in North Queensland as described above.

### iGAS case definition

Although iGAS became notifiable in Queensland in 2005, with Notifiable Conditions System (NOCS) data available from 2006 onwards, ICD-10 coding was used throughout to enable comparable case identification across all years. ICD-10 codes used to identify iGAS cases, are listed in Box 1 in Online Resource 2. A single admission was defined as the period from the initial date of hospital admission to the date of death or discharge to the patient's usual place of residence. Readmission was defined as hospital admission within 30 days of discharge to the patient’s usual residence; admissions after 30 days were considered new admissions.

### Statistical analysis

The data analysis was conducted using R version 4.0.3. Socio-demographic characteristics and common risk factors of the study population were assessed, and the incidence of iGAS cases was evaluated. Denominator population data were obtained from the Australian Bureau of Statistics (ABS). The population of the midpoint census year (2011) was used to calculate crude and age-standardised incidence rates for the entire period. For temporal trend analyses, population denominators from ABS census years (2006, 2011, 2016 and 2021) were obtained and intercensal populations were estimated by linear extrapolation. Postcode-level population data were used for spatial analyses (Fig. 5), while age-, sex-, and Indigenous status–stratified populations were obtained for analyses presented in Figs. 3 and 4 using the ABS Table Builder tool [13]. Age- and sex-standardised incidence rates were calculated using the 30 June 2001 Australian standard population [14]. Disease counts and incidence were analysed across age, sex, and Indigenous status categories over the study period. The incidences were calculated to postcode level, and the geographical distribution was mapped using the relevant shapefiles obtained from ABS. Spatial clustering of iGAS incidence was assessed using the global Moran’s I statistic implemented in the spdep package (version 4.0.3). The total study period was divided into four distinct time intervals to account for changes in postcode geographic boundaries throughout the study period and to facilitate the visualisation of trends in disease incidence across different geographic areas. Year 2020 was excluded from this analysis as it has been well documented that there was a significant decline in iGAS cases in Australia as a result of public health measures to slow the spread of COVID-19 [15–17]. Spatial weights were constructed using postcode boundary shapefiles via the poly2nb() function with Queen contiguity, and Moran’s I statistics were computed using the moran.test() function within the same package. Postcode-level incidence data were linked to the corresponding boundary polygons to define spatial relationships. A significant positive Moran’s I indicated spatial clustering of similar incidence values across neighbouring postcodes [18]. A Generalized Additive Model (GAM) was fitted to examine associations between event counts and calendar year, age, sex, and Indigenous status, with population included as an offset. Calendar year was included to assess temporal variation in incidence, while age was modelled non-linearly due to the expected non-linear relationship between age and iGAS risk. Sex and Indigenous status were included a priori based on established epidemiological differences in iGAS incidence. Population size was incorporated as an offset to estimate incidence rates. The GAM approach enabled estimation of adjusted incidence rate ratios (IRRs) while flexibly modelling non-linear relationships between age and calendar year and controlling for sex and Indigenous status. Thin plate regression splines were used for the smooth terms (s(start_year) and s(age)) as these are the default basis in the mgcv (version 4.0.3) package in R and provide flexible modelling of non-linear relationships while avoiding the need to pre-specify knot locations. The degree of smoothness is estimated from the data during model fitting. The model was fitted using the mgcv package (version 4.0.3) in R which is expressed as below [19]. GAM was used to assess temporal trends within the observed study period only. Model fit was assessed using gam.check() and concurvity() diagnostics from the mgcv package. The adequacy of spline basis dimensions was evaluated using the k-index test, and residual diagnostics were reviewed to assess model performance.

$$ \begin{gathered} {\mathrm{log}}\left( {\text{Event count}} \right){\mkern 1mu} { = }{\mkern 1mu} {\mathrm{s}}\left( {{\mathrm{Start}}\_{\mathrm{year}}} \right){\mkern 1mu} {\text{ + s (Age) }} \hfill \\ { + }\beta_{{1}} {\mkern 1mu} \cdot Sex{\mkern 1mu} + {\mkern 1mu} \beta_{2} {\text{ Indigenous status}}{\mkern 1mu} { + }{\mkern 1mu} {\mathrm{log(Population)}} \hfill \\ \end{gathered} $$

where s() represents a smooth spline function, and log(population) is an offset.

### Ethical approval

The project has obtained the ethics approval from The Townsville Hospital and Health Service Human Research Ethics Committee (HREC Reference number: HREC/16/QTHS/221) and Public Health Act approval to conduct this study. In addition, the ethics approval was obtained by the James Cook University Human Research Ethics Committee (HREC) to ensure research is conducted rigorously, respectfully, and in alignment with Indigenous rights and ethical standards.

## Results

### Characteristics of the population

During the 21-year study period, a total of 933 invasive disease events were reported among 870 individual patients. The mean age of the population was 45 years (SD = 24), ranging from < 1 to 93 years. Indigenous individuals were comparatively younger, with a mean age of 42 years (SD = 22; range: < 1 to 89 years), compared to non-Indigenous individuals whose mean age was 48 years (SD = 26; range: < 1 to 93 years). Males comprised 54.9% (n = 478) of the study population. The largest proportion of cases occurred among individuals aged 50–59 years (17.2%). Children aged under 10 years accounted for 11.7% of cases. Nearly half of the patients identified as Aboriginal and or Torres Strait Islander (45.6%). Most patients resided in Outer Regional areas (65.9%). In terms of socioeconomic status, the majority of patients lived in relatively (47.9%) or most (22.5%) disadvantageous areas, with fewer patients residing in more advantaged areas. Table 1 summarises the sociodemographic characteristics of the study population.

**Table 1:** Characteristics of the study population (n = 870)

Characteristic	Number (%)
Total number of patients	870
Sex	
Male	478 (54.9)
Female	392 (45.1)
Age group (Years)	
< 1	23(2.6)
1–9	79 (9.1)
10–19	49 (5.6)
20–29	106 (12.2)
30–39	84 (9.7)
40–49	112 (12.9)
50–59	150 (17.2)
60–69	110 (12.6)
70–79	89 (10.2)
≥ 80	68 (7.8)
Indigenous status	
Aboriginal and / or Torres strait Islander	397(45.6)
Neither aboriginal nor Torres strait Islander	466 (53.6)
Not stated	7 (0.8)
Remoteness of the residence of patients	
Major cities	0 (0)
Inner regional	23 (2.6)
Outer regional	573 (65.9)
Remote	125 (14.4)
Very remote	149 (17.1)
Socio-economic indexes for areas quantile	
Most disadvantageous	196 (22.5)
Relatively disadvantageous	417 (47.9)
Averagely advantageous	94 (11)
Advantageous	145 (16.7)
Least disadvantageous	18 (2.1)

### Disease incidence

**Fig. 2:**
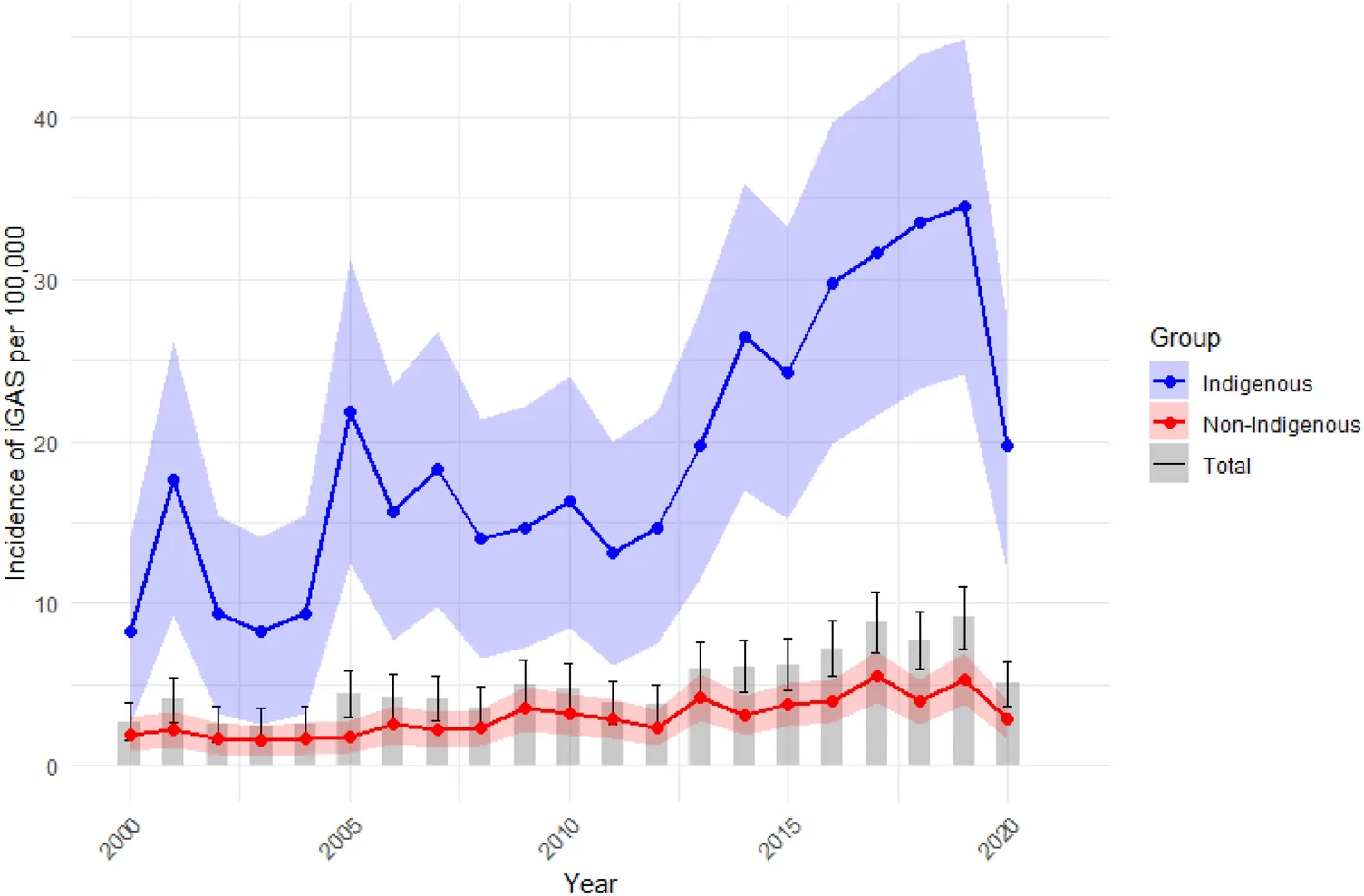
Incidence of iGAS disease, 2000–2020

The average annualised crude incidence was 19.7 per 100,000 population among Indigenous Australians and 3.0 per 100,000 population among non-Indigenous Australians. After age standardisation, the incidence remained substantially higher among Indigenous Australians (29.5 per 100,000) compared with non-Indigenous Australians (3.0 per 100,000). The average annualised incidence rate for males was 3.48 per 100,000 person-years (95% CI: 1.88–5.88) among non-Indigenous individuals, and 20.84 per 100,000 (95% CI: 10.38–37.33) among Indigenous individuals. For females, the corresponding rates were 2.57 per 100,000 (95% CI: 1.20–4.80) for non-Indigenous individuals and 18.66 per 100,000 (95% CI: 8.98–34.21) for Indigenous individuals. The age-standardised incidence rate for males was 3.30 per 100,000 (95% CI: 2.95–3.68) for non-Indigenous and 31.47 per 100,000 (95% CI: 30.36–32.61) for Indigenous individuals. Among females, the age-standardised rates were 2.64 per 100,000 (95% CI: 2.32–2.98) for non-Indigenous and 27.36 per 100,000 (95% CI: 26.33–28.42) for Indigenous individuals (Online Resource 1). Figure 2 shows the total disease incidence trend (with 95% confidence intervals) across the study period and for Indigenous and non-Indigenous separately. Overall incidence rose from fewer than 3 cases per 100,000 person-years in 2000 to a peak of 9 cases per 100,000 person-years in 2019.

**Fig. 3:**
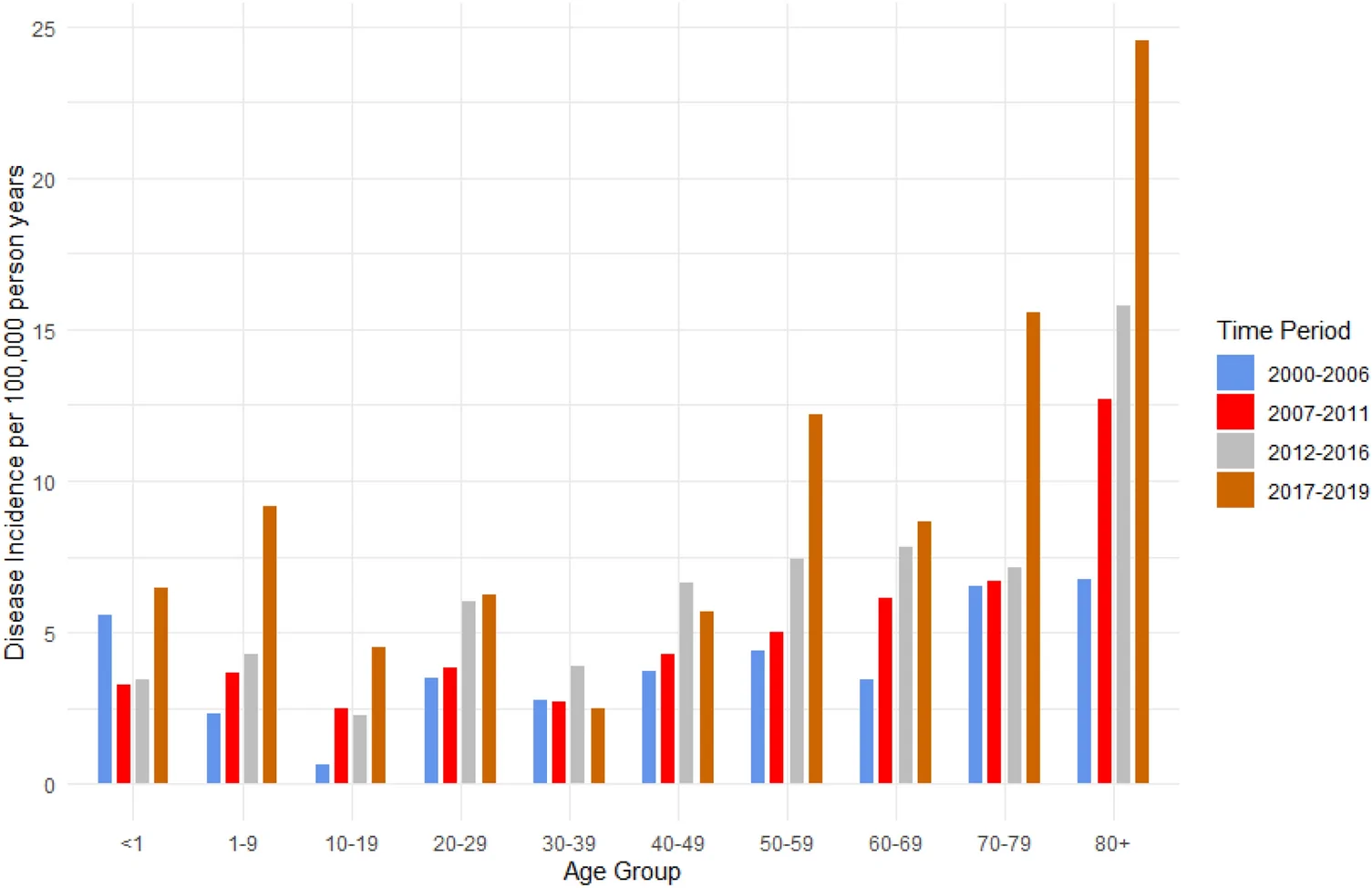
Disease incidence by age group and time period

Overall, incidence increased over time, with a greater disease burden observed among children and older adults (Fig. 3). Across ethnic and sex categories, the incidence of the disease peaked in the 50–59-year age group with a marked decline thereafter, particularly among Indigenous males and females, probably due to the smaller number of older populations (Online Resource 2 Fig. 1). However, the disease incidence was highest among the elderly. Year 2020 was excluded from this analysis for the same reason mentioned above.

According to the negative binomial generalized additive model (GAM) male sex was significantly associated with higher incidence rates of 28% compared with females (IRR = 1.28, SE = 0.073, p < 0.001). Indigenous status was also strongly associated with increased event rates (IRR = 8.97, SE = 0.076, p < 0.001), compared to non-Indigenous group (Table 2).

**Table 2:** Negative binomial GAM examining factors associated with incidence rate

Variable	Estimate (β)	SE	IRR (expβ)	p-value
Intercept	− 10.726	0.070	–	< 0.001
Male sex	0.248	0.073	1.28	< 0.001
Indigenous group	2.193	0.076	8.97	< 0.001

Smooth terms						
Smooth term		edf		χ^2^		p-value
Start year	8.04		129.0		< 0.001	
Age	7.45		304.5		< 0.001	

**Fig. 4:**
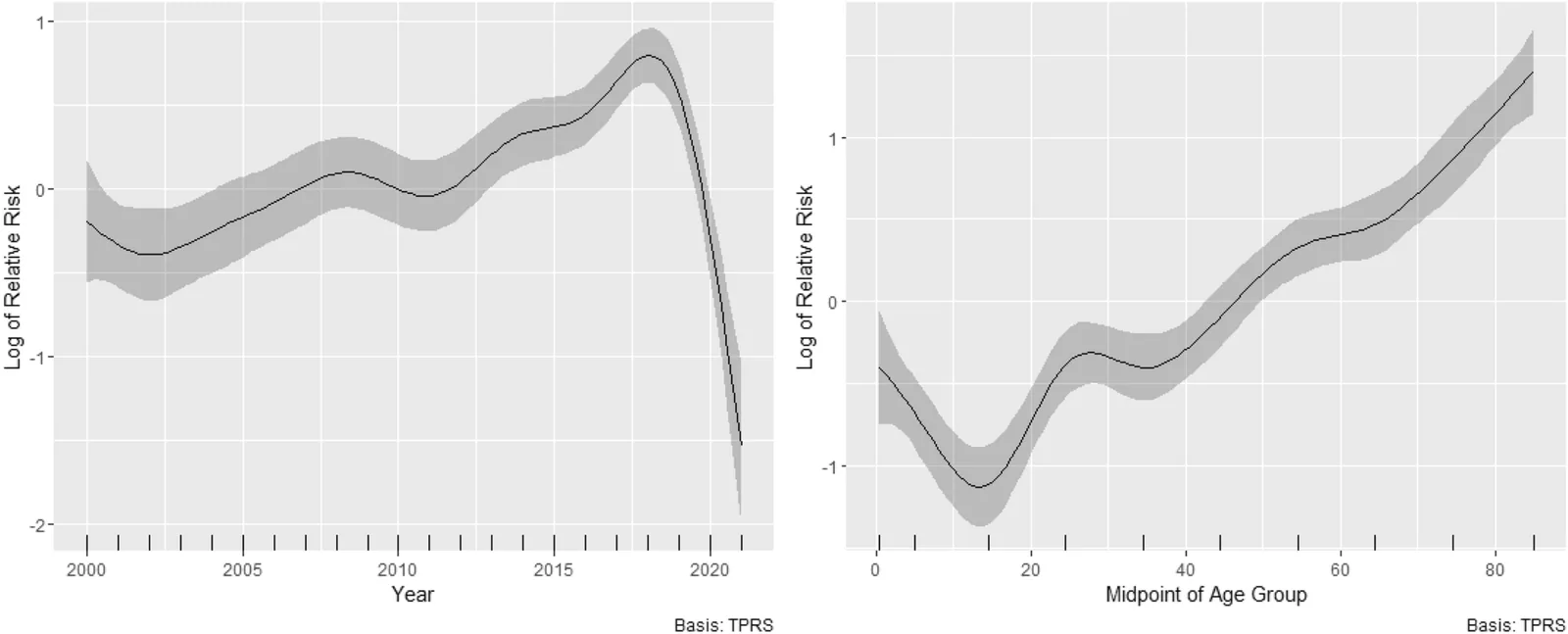
Smooth function of year and age (taken as mid point of each age group)

Both smooth terms demonstrated significant nonlinear associations with event rates. Calendar year showed a significant nonlinear effect (edf = 8.04, χ^2^ = 129.0, p < 0.001), indicating temporal variation in iGAS incidence over the study period. Incidence increased between 2010 and 2018, followed by a marked decline in 2020, coinciding with the period of COVID-19-related public health measures (Fig. 4A). Age also demonstrated a significant nonlinear association with event rates (edf = 7.45, χ^2^ = 304.5, p < 0.001), with incidence rates increasing with age, particularly from midlife onward (Fig. 4B). Overall, the model explained 55.5% of the deviance, with an adjusted R^2^ of 0.261, indicating a moderate level of explanatory performance. We assessed GAM model adequacy using diagnostic functions implemented in the mgcv package, including convergence assessment, basis dimension checking, residual diagnostics, and concavity assessment. The model showed full convergence using REML (Residual Maximum Likelihood) estimation. The basis dimension checks indicated adequate spline complexity, with no evidence that the basis dimensions were insufficient (s(start_year): k-index = 1.22, p = 1.00; s(mid_point_of_age): k-index = 1.26, p = 1.00). Concurvity diagnostics did not indicate problematic relationships between model terms. These diagnostics have been summarised in the Tables 1 and 2 of Online Resource 2.

### Geographical distribution

**Fig. 5:**
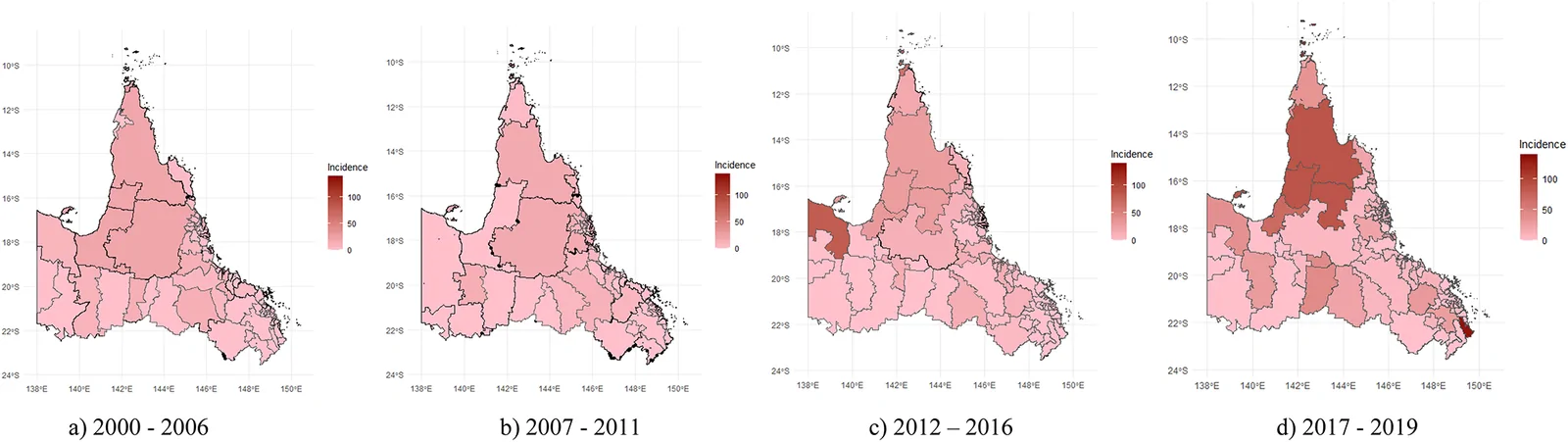
Geographical distribution of iGAS incidence, 2000–2019

The spatial distribution of iGAS incidence over the study period is shown in Fig. 5. The map showed an increase in the incidence of iGAS with time, especially in the remote and very remote areas where there is also a high Indigenous population. Global Moran’s I analysis demonstrated variation in the spatial distribution of iGAS incidence across the four study periods. Significant positive spatial autocorrelation was observed in 2006 (Moran’s I = 0.425, p < 0.001), 2016 (Moran’s I = 0.146, p = 0.005), and 2021 (Moran’s I = 0.196, p < 0.001), indicating clustering of similar incidence values across neighbouring postcodes. No significant spatial autocorrelation was observed in 2011 (Moran’s I = -0.014, p = 0.580), suggesting a random spatial distribution during this period.

### Clinical manifestations and potential risk factors

The most frequently recorded ICD-10 diagnosis was sepsis due to GAS, accounting for 564 cases (60.5%), followed by osteomyelitis (211 cases, 22.6%) and arthritis (104 cases, 11.2%) (Table 3). Additionally, there were 17 cases (1.8%) of streptococcal toxic shock syndrome (STSS) and 29 cases (3.3%) of necrotising fasciitis (NF). In further analysis we presented the characteristics of NF and STSS cases separately because these are severe manifestations of iGAS with important clinical implications (Table 4). Patients with NF were older than those with STSS, with a mean age of 54.7 years compared with 33.7 years. Males comprised the majority of cases in both groups (62% in NF and 53% in STSS). Indigenous patients accounted for 41% of NF cases and 59% of STSS cases. Disease type by age group, sex and Indigenous status is depicted in Fig. 2 in Online Resource 2.

**Table 3:** Clinical manifestations (percentages do not add up to 100% because individuals may have multiple clinical manifestations)

Clinical manifestation	Number of events (% out of all events)
Total number of events	933 (100)
Sepsis	564 (60.5)
Osteomyelitis	211 (22.6)
Arthritis	104 (11.2)
Puerperal sepsis	62 (6.7)
Necrotizing fasciitis	29 (3.3)
Pyothorax with/ without fistula	25 (2.7)
Pneumonia	24 (2.6)
Toxic shock syndrome	17 (1.8)
Retropharyngeal and parapharyngeal abscess	14 (1.5)
Meningitis	3 (0.3)
Sepsis of newborn	2 (0.2)

**Table 4:** Demographic characteristics of patients with NF and STSS

	NF (%)	STSS (%)
Number (% of all iGAS cases)	29 (3.3)	17 (1.8)
Mean age (years)	54.7 years	33.7 years
Male (%)	18 (62)	9 (53)
Female (%)	11 (38)	8 (47)
Indigenous (%)	12 (41)	10 (59)
Non-Indigenous (%)	17 (59)	7 (41)

The distribution of comorbidities and behavioural risk factors among Indigenous and non-Indigenous individuals with iGAS infection, including statistical comparisons between groups using chi-square tests, with Fisher’s exact test applied where appropriate is presented in Table 5 (ICD-10 codes of relevant risk factors are shown in Online Resource 2 Table 3). Statistically significant differences were observed for most risk factors (p < 0.001). Drug use was uncommon and similar in both groups, while varicella, HIV infection, and chemotherapy use were rare across the cohort.

**Table 5:** Distribution of comorbidities and behavioural risk factors among Indigenous and non-Indigenous individuals with iGAS infection

Risk factor	Indigenous	Non-indigenous	Total	P value
Total number	397	466	870	
Skin infections	297 (74.8%)	276 (59.2%)	573 (66.4%)	< 0.001
Diabetes	214 (54.2%)	108 (22.9%)	322 (37.3%)	< 0.001
Cardiac disease	192 (48.4%)	204 (43.8%)	396 (45.9%)	< 0.001
Renal failure	221 (55.7%)	167 (35.8%)	388 (44.9%)	< 0.001
Smoking	270 (68.0%)	238 (51.1%)	508 (58.9%)	< 0.001
Harmful alcohol consumption	138 (34.8%)	81 (17.4%)	219 (25.4%)	< 0.001
Drug use	20(5.0%)	21 (4.5%)	41 (4.8%)	0.22
Open wounds	162 (40.8%)	130 (27.9%)	292 (33.8%)	< 0.001
Neoplasms	41 (10.3%)	92 (19.7%)	133 (15.4%)	< 0.001
Renal dialysis	73 (18.4%)	13 (2.8%)	86 (10.0%)	< 0.001
Varicella	2 (0.5%)	3 (0.6%)	5 (0.6%)	0.94
HIV	0(0%)	0(0%)	0(0%)	–
Chemotherapy use	10(02.5%)	19(04.1%)	29 (3.4%)	0.4

^*^Seven participants whose Indigenous status was not mentioned were excluded from this analysis

## Discussion

We identified a substantial burden of iGAS disease in Northern Queensland, Australia, with a marked increase in annual incidence over the study period, particularly among Indigenous populations. Among non-Indigenous individuals, incidence remained below 7 cases per 100,000 person-years throughout the study period, with only a modest upward trend. In contrast, incidence among Indigenous populations increased markedly, from fewer than 3 cases per 100,000 person-years in 2000 to 35 cases per 100,000 person-years by 2019. Similar increasing trends have been reported in studies from other parts of Australia during the same period [20–22], as well as in international studies conducted over comparable time frames [23, 24]. The observed increase in iGAS incidence over time may reflect a combination of changing population risk profiles, rising prevalence of comorbidities, climatic factors influencing bacterial transmission and improvements in disease detection and surveillance. A notable decline in iGAS incidence was observed in our study in 2020 among both Indigenous and non-Indigenous populations. One possible contributor may be public health measures introduced in response to the COVID-19 pandemic, including increased hygiene practices, social distancing, and heightened public awareness of communicable diseases which is well documented in other studies [15–17]. Further investigation into disease patterns beyond 2020 would provide valuable insights into the lasting impact of these interventions.

In our study, the crude and age-standardised incidence of iGAS among Indigenous Australians was 6.5-fold and 9.8-fold higher, respectively, than that observed among non-Indigenous Australians. Age-standardised incidence rates were calculated using the 2001 Australian standard population to enable comparison between Indigenous and non-Indigenous populations. However, we acknowledge that age-standardised rates are relative measures and may be influenced by the choice of standard population, particularly when comparing populations with different age structures. Therefore, these estimates should be interpreted alongside crude incidence rates, which reflect the observed disease burden within each population [25]. A prior study conducted in the same region reported that the incidence of iGAS was approximately eight times higher among Indigenous populations compared to non-Indigenous [26]. This pattern is consistent with findings from a recent meta-analysis, which highlighted significantly higher iGAS risk among Indigenous peoples across Australia [6]. These findings may reflect disparities in social determinants of health, access to healthcare and higher prevalence of comorbidities and behavioural risk factors among Indigenous patients, as observed in this study. Similar disparities have been documented with studies outside Australia reporting elevated iGAS incidence among Indigenous populations [27, 28].

Spatial mapping showed a clear northward trend in iGAS incidence, with Far North Queensland experiencing a disproportionately high and increasing burden, particularly in remote regions with large Indigenous populations. Significant spatial clustering indicated that disease incidence was geographically concentrated rather than randomly distributed. To our knowledge, this is the first Australian study to apply spatial autocorrelation analysis to iGAS incidence, highlighting important geographic patterns that may reflect disparities in social determinants of health, healthcare access, environmental conditions, and underlying risk factors. These findings support the need for geographically targeted public health interventions in high-incidence regions.

In our study, the most frequently recorded diagnosis was sepsis, followed by osteomyelitis and septic arthritis. These findings are consistent with those reported in another Australian study conducted in Victoria [29]. The proportion of STSS cases in our cohort was relatively low, at 1.8%. In contrast, studies conducted across various Australian states and territories between 1991 and 2020 reported higher STSS prevalence rates, ranging from 6.6% to 16.5% [8, 26, 30–33]. NF was identified in 3.3% of cases in our study, which falls within the range reported in previous studies (0.26% to 11%) in Australia from 1998 to 2017 [29, 30, 32, 33]. Further analysis of STSS and NF cases revealed distinct demographic patterns: NF was more common among middle-aged males, whereas STSS was more frequently observed in younger males. NF was more prevalent among non-Indigenous whereas STSS was more prevalent among Indigenous. A study conducted in the Northern Territory between 2011 and 2013 reported no significant difference in STSS incidence between Indigenous and non-Indigenous populations [8]. The lower proportion of STSS in our cohort compared with earlier Australian studies may reflect temporal and geographic variation in circulating *Streptococcus pyogenes emm* types, which are strongly associated with specific virulent strains. However, emm typing data were not available, limiting molecular comparison. Improved clinical recognition, earlier treatment, and differences in coding and case ascertainment may also contribute. Further research incorporating molecular surveillance is needed to better understand STSS variation across populations and over time.

The disease incidence of iGAS consistently increased with age. Among non-Indigenous individuals, incidence remained below 1 case per 100,000 person-years across all age categories with a slight increase after the age of 60 years. Conversely, Indigenous populations exhibited a high incidence in the < 1 year age group, which then decreased during the 1 to 19 years age range, before rising sharply in older age groups. Similar disease patterns have been reported in previous studies conducted in Western Australia and Victoria [20, 29, 33]. Many studies identified older age as a risk factor for iGAS [29, 30, 34]. However, a study conducted in Northern Territory reported a differing pattern, with peak incidence occurring in middle-aged populations followed by a subsequent decline [8]. These findings suggest that while age is a well-established risk factor for iGAS infection, other factors, particularly ethnicity and comorbidities also play a significant and contributory role.

Our Generalised Linear Model identified male sex and Indigenous status as significant risk factors for iGAS, consistent with findings from previous studies [22, 35]. This may be attributable to the higher prevalence of underlying risk factors among men and Indigenous populations.

Despite the strengths of this study, including comprehensive coverage of a large geographic area and a 21-year longitudinal dataset, a few limitations should be acknowledged. iGAS cases were identified using ICD-10 codes, which may have lower specificity compared with notification-based surveillance criteria; however, this approach was necessary to ensure consistent case ascertainment across the full study period. In addition, the use of postcode-level geographic data, with changing boundaries over time, complicated spatial and temporal comparisons. Future studies should consider more stable geographic units, such as Statistical Area levels, to improve spatial accuracy. A larger dataset would also enable more detailed analysis of severe iGAS manifestations such as STSS and NF, which were limited by small case numbers.

In conclusion, this study demonstrates a clear increase in iGAS incidence in Northern Queensland over the 21-year period, with a notably higher rise among Indigenous populations. Evidence of geographic clustering and evolving clinical patterns was also observed. The findings of this study may inform the strategic allocation of healthcare resources and support the identification of high-risk populations and priority areas for targeted disease prevention efforts.

## Supplementary Information

Below is the link to the electronic supplementary material.

Online Resource 1 (XLSX 16 KB)

Online Resource 2 (DOCX 81 KB)

## Data Availability

The data underlying this study are available from the corresponding author upon reasonable request and with appropriate approvals.
