# Supplementary material for: Emerging epidemiology of invasive group a Streptococcus in tropical Australia: a 21-year population-based data linkage study
Source: Infection. 2026 Aug 25;54(5):2681–90. doi: 10.1007/s15010-026-02904-w (PMC13727874; doi:10.1007/s15010-026-02904-w)
Supplement: Supplementary file 1 [file 15010_2026_2904_MOESM2_ESM.docx]

**Online Resource**

**Box 1: The ICD-10 codes used to identify the confirmed and probable cases of iGAS**

A 400 - Sepsis due to streptococcus, group A

B 950 - Streptococcus, group A, as the cause of diseases classified elsewhere together with one of the following codes

A 483 - Toxic shock syndrome

G002 - Streptococcal meningitis

J154 - Pneumonia due to other streptococci

J 390 - Retropharyngeal and parapharyngeal abscess

J 860 - Pyothorax with fistula

J 869 - Pyothorax without fistula

M 002 (Other streptococcal arthritis and polyarthritis) which includes M 0020 to M 0029 which further specifies the disease site

M 726 - Necrotizing fasciitis

M 86 (Osteomyelitis) which includes M 860 to M 869 which further classifies the disease type or site

M 462 to M 4628 - Osteomyelitis of vertebra

M 463 to M 4639 - Infection of intervertebral disc (pyogenic)

M 465 to M4659 - Other infective spondylopathies

O 85 - Puerperal sepsis

P 361 - Sepsis of newborn due to other and unspecified streptococci

P 3610 - Sepsis of newborn due to unspecified streptococci

P 3619 - Sepsis of newborn due to other streptococci

0 8612 - Endometritis following delivery

**Figure 1: Disease incidence and count by age, sex and Indigenous status (2000-2019)**

**Table 1 : Generalized additive model (GAM) convergence and basis dimension assessment**

| **Model diagnostic** | **Result** |
| --- | --- |
| **Estimation method** | Restricted maximum likelihood (REML) |
| **Optimisation method** | Outer Newton optimiser |
| **Model convergence** | Full convergence achieved after 5 iterations |
| **Gradient range** | −0.00064 to 0.00031 |
| **Hessian** | Positive definite |
| **Model rank** | 20 / 21 |
| **Deviance explained** | 55.5% |
| **Adjusted R²** | 0.261 |

**Table 2: Basis dimension checking for smooth terms in the GAM**

| **Smooth term** | **Basis dimension (k')** | **Estimated degrees of freedom (edf)** | **k-index** | **p-value** |
| --- | --- | --- | --- | --- |
| s(start_year) | 9.00 | 8.02 | 1.22 | 1.00 |
| s(mid_point_of_age) | 9.00 | 6.41 | 1.26 | 1.00 |

**Figure 2: disease count by iGAS type, age group, sex and Indigenous status**

**Table 3: ICD-10-AM codes of relevant risk factors (‘x’ indicates a further subdivision of the specific disease type)**

| **Risk factor** | **ICD-10-AM codes** | **Description** |
| --- | --- | --- |
| Skin infections | L00xx – L08x | Skin infections |
| Renal failure | N17 – N17.x  N18 – N18.xx  N19  I120  I131  I132  P960 | Acute kidney failure  Chronic kidney disease  Unspecified kidney failure  Hypertensive kidney disease with kidney failure  Hypertensive heart and kidney disease with kidney failure  Hypertensive heart and kidney disease with both (congestive) heart failure and kidney failure  Congenital kidney failure |
| Cardiac disease | I01-I01.x  I02.0  I05x – I09x  I10 – I15x  I20 – I25x  I26x – I28x  130x – 152x | Rheumatic fever with heart involvement  Rheumatic chorea with heart involvement  Rheumatic Heart Disease  Hypertensive Disease  Ischaemic Heart Disease  Pulmonary embolism, other pulmonary heart disease, other diseases of pulmonary vessels  Other diseases of the heart including pericardial disease, endocarditis, valve disorders, myocarditis, cardiomyopathy, conduction disorders, cardiac arrest, arrhythmias, heart failure |
| Renal dialysis | Y841  Z49  Z490  Z491  Z492  Z992 | Kidney dialysis  Care involving dialysis  Preparatory care for dialysis  Extracorporeal dialysis  Other dialysis  Dependence on kidney dialysis |
| Smoking | Z720  Z8643 | Tobacco use, current  Personal history of tobacco use disorder |
| Diabetes | E10xx  E11xx  E12xx  E13xx  E14xx | Type 1 diabetes mellitus  Type 2 diabetes mellitus  Malnutrition-related diabetes mellitus  Other specified diabetes mellitus  Unspecified diabetes mellitus |
| Neoplasms | Cxxxx  D0xx  D37xx – D489 | Malignant neoplasms  Carcinoma and melanoma  Neoplasms of uncertain or unknown behaviour |
| Drug use | Z722  Z8642 | Drug use  Personal history of drug use disorder |
| Varicella | B01.xx | Varicella (Chickenpox) |
| HIV | B20  B21  B22  B23  B230  B238  B24 | Human immunodeficiency virus [HIV] disease resulting in infectious and parasitic diseases  Human immunodeficiency virus [HIV] disease resulting in malignant neoplasms  Human immunodeficiency virus [HIV] disease resulting in other specified diseases  Human immunodeficiency virus [HIV] disease resulting in other conditions  Acute HIV infection syndrome  HIV disease resulting in other specified conditions  Unspecified human immunodeficiency virus [HIV] disease |
| Chemotherapy use | Z511  Z512  Z926  Z082 | Pharmacotherapy session for neoplasm  Other chemotherapy  Personal history of pharmacotherapy for neoplastic disease  Follow-up examination after pharmacotherapy for malignant neoplasm |
| Alcohol use | Z721  Z8641  F10 – F109 | Alcohol use  Personal history of alcohol use disorder  Mental and behavioural disorders due to alcohol use |
